# The Ingestion of Dietary Prebiotic Alternatives during Lactation Promotes Intestinal Health by Modulation of Gut Microbiota

**DOI:** 10.4014/jmb.2208.08017

**Published:** 2022-10-24

**Authors:** Sangdon Ryu, Jeong Jae Lee, Daye Mun, Soo Rin Kim, Jeehwan Choe, Minho Song, Younghoon Kim

**Affiliations:** 1 Department of Agricultural Biotechnology and Research Institute of Agriculture and Life Sciences, Seoul National University, Seoul 08826, Republic of Korea; 2 Institute of Agricultural Science and Technology, Kyungpook National University, Daegu 41566, Republic of Korea; 3 School of Food Science and Biotechnology, Kyungpook National University, Daegu 41566, Republic of Korea; 4 Major of Beef Science, Department of Livestock, Korea National University of Agriculture and Fisheries, Jeonju 54874, Republic of Korea; 5 Division of Animal and Dairy Science, Chungnam National University, Daejeon 34134, Republic of Korea

**Keywords:** Palm kernel expeller, gut microbiota, *Lactobacillus*, *Prevotella*

## Abstract

Palm kernel expeller (PKE), a by-product of palm oil extraction, contains higher amounts of fiber than corn and soybean meal, but offers low energy density, protein value, and amino acid (AA) composition, limiting its use for swine. Recently however, it was reported that dietary fiber has a positive effect on the gut microbiota of the host, and therefore it is necessary to study the effect of PKE feeding on the intestinal microbiota of swine. In this study, we investigated the effects of supplementation with PKE in lactation diets on the gut microbiota composition of lactating sows and their litters. A total of 12 sows were randomly assigned to two dietary treatment groups in a completely randomized design. The treatments were a diet based on corn-soybean meal (CON) and CON supplemented with 20% of PKE. Sow and piglet fecal samples were collected before farrowing, on days 7 and 28 (weaning) after farrowing, and on days 7 and 28 (weaning) after farrowing, respectively, to verify gut microbiota composition by pyrosequencing analysis. The beta-diversity result showed a significant difference only in weaning-stage piglets, but dietary PKE altered the gut microbiota in sows by increasing the abundance of *Lactobacillus* compared with CON. In piglets, dietary PKE decreased the abundance of opportunistic pathogen *Proteus* and increased the abundance of potentially beneficial bacteria, such as *Prevotellaceae* and *Prevotella*. Our results can be helpful in developing feeding strategies and support the beneficial effects of dietary PKE to improve the gut health of animals.

## Introduction

In recent years, along with a worldwide increase in the production of biofuels, the prices of materials that have been traditionally used as stock feed, such as corn and soybean meal, have been steadily rising [1]. The rising cost of these ingredients has led to the increasing use of cheap by-products as substitutes for grains such as corn and soybean meal, and these have become an important part of swine diets [2]. Among the various types of by-products, palm kernel by-products, such as palm kernel expeller (PKE) and palm kernel meal (PKM) in particular, have been mainly used in ruminant and poultry feeds [3, 4], and not swine feeds, because they have a higher fiber content than corn and soybean meal, but a lower energy density, protein value, and poor amino acid (AA) composition [5]. However, the practice of adding antibiotics to livestock feed has been phased out over recent years in the livestock industries of most developed countries, and as research on gut microbiota has expanded, interest in the use of dietary fiber to improve performance and immune response by regulating the gut microbiota is increasing [6, 7].

In the field of animal science, study into intestinal microbial ecology is conducted through various nutritional strategies, and it has become recognized that this microbial community, where various bacterial species present in the gastrointestinal tract form a huge ecology, has a significant impact on the health of the host [8, 9]. Dietary components contained in feed can have a direct effect on the intestinal environment, and in particular, dietary fiber can change not only the composition of various microorganisms living in the digestive tract, but also other conditions of the host’s intestinal health [10-12].

In a previous study, we showed that the addition of 20% palm kernel expeller to a diet based on corn and soybean meal in lactating sows did not adversely affect productivity, nutrient digestion, and white blood cell (WBC) changes [13]. In relation to the importance of intestinal microbiota with respect to the availability of dietary fiber, one study found that adding PKM to a chicken diet increased *Lactobacillus* spp. and *Streptococcus* spp. in the cecum and ileum [14]. However, little information is available on the gut microbiome of lactating sows fed PKE. Based on previously reported studies, we hypothesized that adding PKE with high fiber content to the diet of sows would affect the gut microbiome and health of sows. In addition, we further investigated whether it could affect the gut microbiota of piglets born from sows fed with PKE.

Therefore, our aim in this study was to examine the effect of palm kernel expeller on fecal microbial diversity and bacterial community composition of lactating sows and their litters using pyrosequencing of the 16S rRNA genes.

## Materials and Methods

### Experimental Design, Animals, Diets, and Sample Collection

A total of 12 lactating sows (Landrace x Yorkshire; 200 ± 12 kg of average BW; 2.5 of average parity) were used in this experiment. Sows were randomly assigned to dietary treatments and fed the dietary treatments from 30 days before farrowing to weaning. Dietary treatments were a typical lactation diet based on corn and soybean meal (CON) and CON added with 20% of PKE (60.9% insoluble dietary fiber + 2.6% soluble dietary fiber). The dietary treatments were formulated to meet or exceed the National Research Council (NRC) estimates of nutrient requirements of lactating sows and to have similar metabolizable energy (ME), crude protein (CP), and AA levels [5, 13]. On 109 days of gestation, sows were moved from gestation crates to farrowing crates equipped with a feeder and waterer in an environmentally controlled farrowing room. Sows were then fed 2.5 kg of the dietary treatments from 109 days of gestation until farrowing and allowed free access to the dietary treatments from farrowing until weaning [15]. Diets and water were freely accessible at all times. Fecal samples of sows were collected on day 109 of gestation, and on days 7 and 28 of lactation. Additionally, fecal samples of their litters were collected on days 7 and 28 of lactation [15, 16]. The feces samples were collected by rectal palpation from three randomly selected sows in each group and two of their randomly selected piglets (1 male and 1 female).

### DNA Extraction, Sequencing, and Metagenomic Analysis

DNA was extracted from fecal samples using the Powerfood Microbial DNA Isolation Kit (Mo Bio Laboratories, Inc., USA) according to the manufacturer’s instructions. The 16S rRNA amplicon sequencing was a performed by Macrogen Inc. (Korea) using a Roche 454 GS-FLX titanium system (454 Life Sciences, USA).

The fasta files and quality data were extracted from the SFF files generated by the Roche 454 GS-FLX titanium system (454 Life Sciences). The reads were further processed using Mothur (v. 1.45.3) [17] following the 454 SOP (https://mothur.org/wiki/454_sop/). Briefly, sequences were grouped according to barcode (1 mismatch allowed) and primer (2 mismatches allowed). Reads were culled if they contained ambiguous characters or homopolymeric runs longer than 8 nucleotides, or failed quality screening (qwindowsize=50, qwindowaverage=35). We aligned the sequences to the Silva database (version 138) [18] and the chimeric sequences were removed by execution of VSEARCH method [19]. Taxonomic classification was analyzed using the RDP classifier [20] and then Chloroplast, Archaea, Mitochondria, and Eukaryota sequences were deleted from further analyses. Low abundance operational taxonomical units (OTUs) and singletons were removed using the Mothur subroutine “split.abund” [21] and sequences with > 97% similarity were classified into OTUs using the distance 0.03 calculation and binned using the opti-clust algorithm.

### Statistical Analysis

For α-diversity, the Chao1 and Shannon indices were performed using Mothur followed by Tukey’s post-hoc analysis if there was a significant difference (*p* < 0.05). For beta-diversity, the Principal Coordinate Analyses (PCoA) was performed based on the Bray-Curtis index and permutational multivariate analysis of variance (PERMANOVA) was conducted to assess the remarkable differences among each sample by mothur. Plots were generated using GraphPad Prism 9.4.1 (USA).

## Results

### Dietary Palm Kernel Expeller Leads to Changes in Microbial Diversity in Lactating Sows and Piglets

To compare changes in microbial composition following feeding with PKE based on a corn-soybean meal diet, we conducted pyrosequencing-based analysis of bacterial 16S rRNA sequence. We investigated the gut microbiota compositions of lactating sows assigned to two dietary treatments (CON+PKE) and their litters at days 7 and 28 during lactation. Initially, a total of 963,923 bacterial sequencing reads and an average read length of 318.937 bp were obtained for 16S rRNA sequences form all fecal samples. The filtered read counts of clustering with 97%similarity were 312,065 for 16S rRNA sequences.

**Fig. 1:**
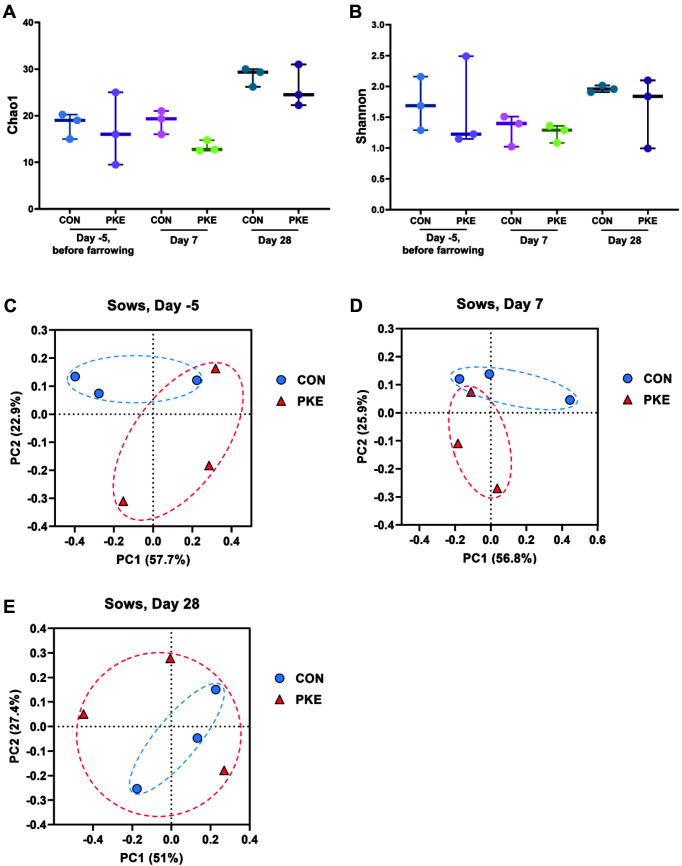
Alpha- and beta-diversity indices on the gut microbiota of lactating sows. Comparison of the alphadiversity indices (**A**) Chao1 and (**B**) Shannon and Principal Co-ordinates Analysis (PCoA) plots of Bray-Curtis index of sow fecal samples at (**C**) day -5 (before farrowing), (**D**) day 7, and (**E**) day 28.

**Fig. 2:**
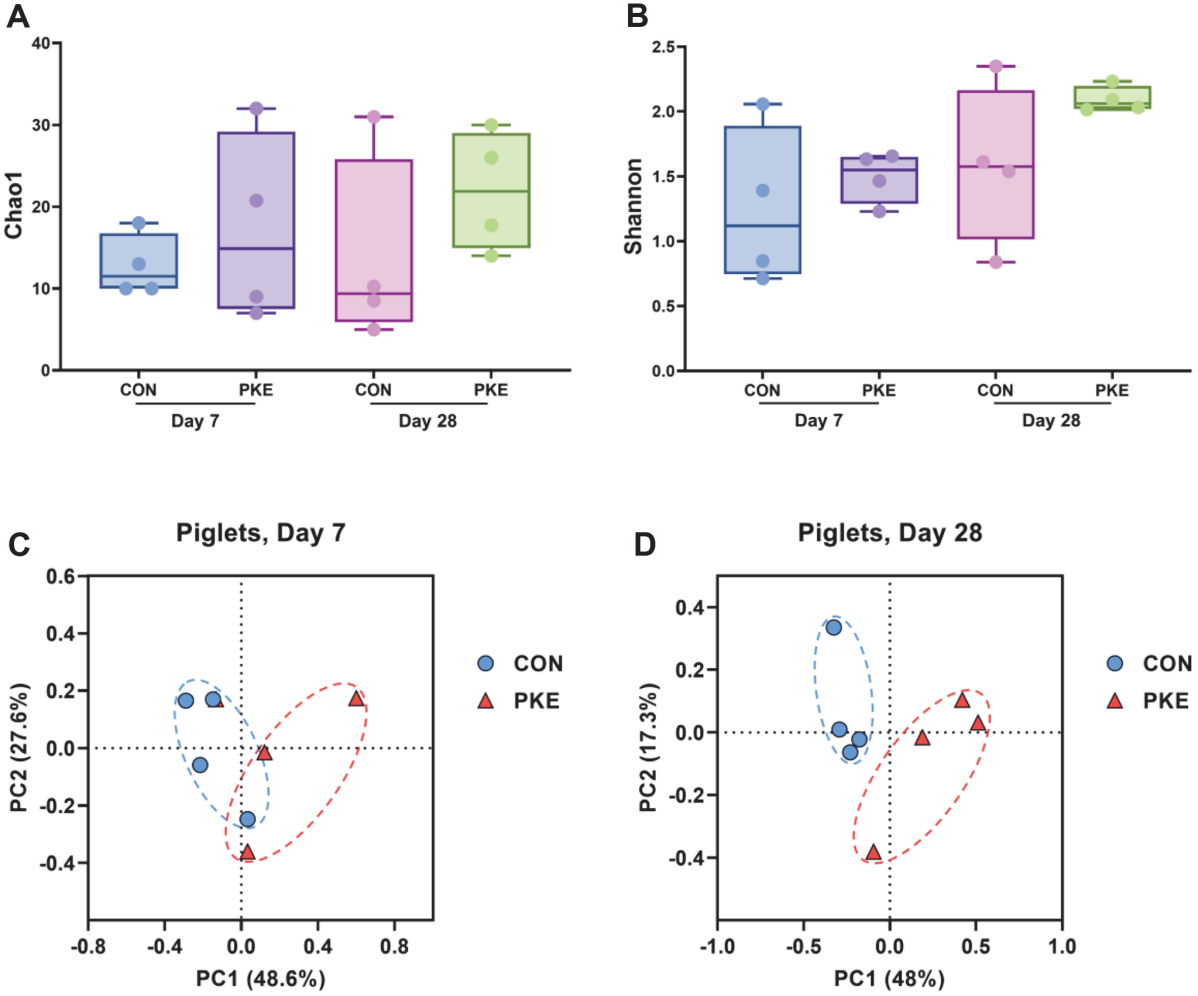
Alpha- and beta-diversity indices on the gut microbiota of piglets. Comparison of the alpha-diversity indices (**A**) Chao1 and (**B**) Shannon and Principal Co-ordinates Analysis (PCoA) plots of Bray-Curtis index of piglet fecal samples at (**D**) day 7 and (**D**) day 28.

The total number of OTUs of lactating sows was higher in PKE (341) compared with CON (318), and like the lactating sows, litters also showed higher levels in PKE (1,073) than in CON (766). The alpha diversity indices were observed using Chao1 and Shannon. In case of lactating sows, the Chao1 index representing the bacterial richness did not differ significantly between the CON and PKE groups at all three time points (Fig. 1A). As with the Chao1 index result, the Shannon index, which provides important data on richness and evenness within the community, did not differ significantly (Fig. 1B). In the piglet group, as in the lactating sow group, there was no significant difference between PKE and CON at both time points in Chao1 (Fig. 2A) and Shannon indices (Fig. 2B), but overall the two alpha-diversity indices were higher in the PKE group.

Next, we examined beta-diversity distances using the Bray-Curtis index. The PCoA plot constructed based on the Bray-Curtis index revealed that the PKE fecal microbiota of piglets separately clustered from that of the CON group that of the CON group at day 28 (Fig. 2D; PERMANOVA, *p* = 0.029). Sows showed no significant difference at all three time points, and piglets showed no statistically significant difference at day 7. These results suggest that supplementation of PKE to lactating sows led to changes in the gut microbiota diversity of weaning piglets.

### Dietary Palm Kernel Expellers Alter Microbial Compositions in Lactating Sows

**Fig. 3:**
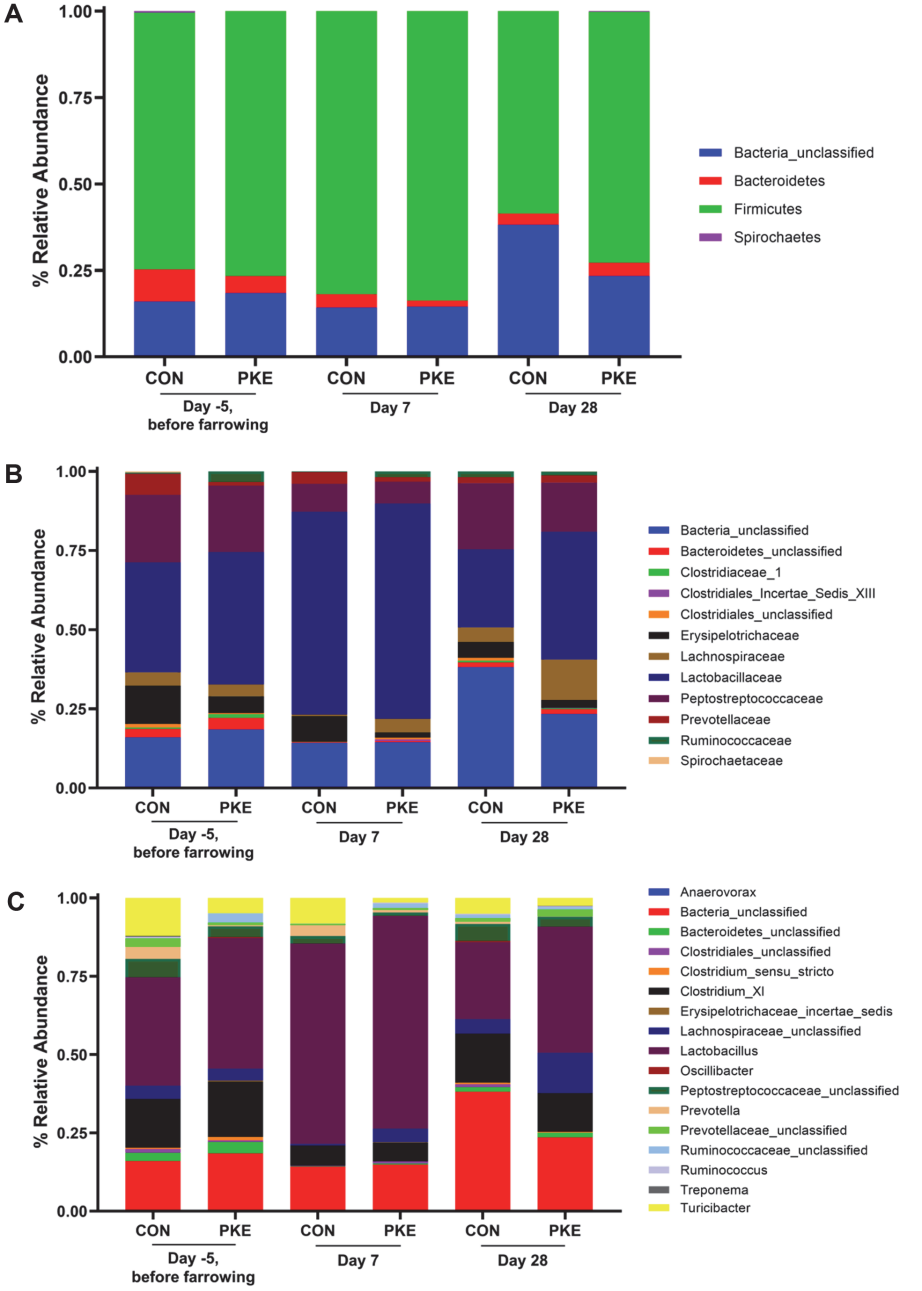
Composition of gut microbiota in lactating sows at day -5 (before farrowing), day 7, and day 28. The composition is made in (**A**) color corresponds to phylum, (**B**) color corresponds to family, and (**C**) color corresponds to genus.

At the phylum level, the predominant bacterial taxa were Firmicutes and Bacteria_unclassified, followed by Bacteroidetes and Spirochaetes in both groups (Fig. 3A). Firmicutes showed the highest abundance in common at all three time points in the CON and PKE groups, and there was no significant change at the phylum level according to fecal sampling time. On day 28, the abundance of Firmicutes tended to be higher in the PKE group than in the CON group (58.52% vs. 72.67% for CON and PKE, respectively) and the abundance of relatively unclassified bacteria showed a higher result in the CON group (38.15% vs. 23.36% for CON and PKE, respectively). At the family level, *Lactobacillaceae*, Bacteria_unclassified, and *Peptostreptococcaceae* were the most abundant bacterial taxa (Fig. 3B). What is noteworthy here is that the abundance of *Lactobacillaceae* at all time points tends to be higher in the PKE group than in the CON group.

At the genus level, the predominant bacterial taxa were *Lactobacillus*, Bacteria_unclassified, and *Clostridium*_XI, followed by *Lachnospiraceae*_unclassified, *Turicibacter*, and *Peptostreptococcaceae*_unclassified in both groups (Fig. 3C). *Lactobacillus* was the most abundant genus in both groups. Supplementation with PKE led to increase in *Lactobacillus* and *Lachnospiraceae*_unclassified. On day 7, the abundance of *Lactobacillus* tended to be slightly higher in the PKE group than in the CON group (64.04% vs. 68.02%). However, on day 28, although the relative abundance in all genera was decreased, it was confirmed that the abundance of *Lactobacillus* increased more in the PKE group than in the CON group (24.63% vs. 40.3%). Additionally, compared to the CON group, it was confirmed that the abundance of *Lachnospiraceae*_unclassified was increased in the PKE group and the proportion of *Turicibacter* and *Peptostreptococcaceae*_unclassified decreased.

### Dietary Palm Kernel Expeller Alters Microbial Compositions in Piglets

**Fig. 4:**
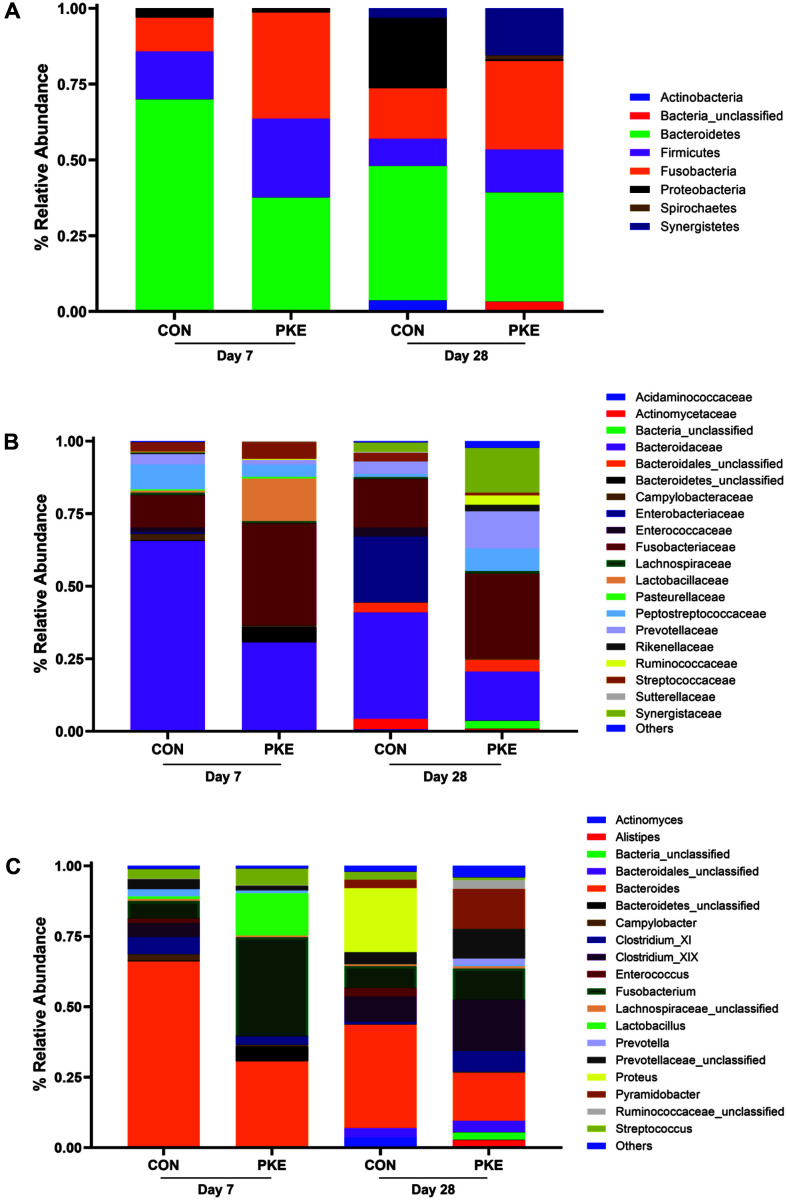
Composition of gut microbiota in piglets at day 7 and day 28. The composition is made in (**A**) color corresponds to phylum, (**B**) color corresponds to family, and (**C**) color corresponds to genus.

At the phylum level, the predominant bacterial taxa were Bacteroidetes, Fusobacteria, and Firmicutes in both groups (Fig. 4A). On day 7, the proportion of Bacteroidetes (70% vs. 37.25%) and Proteobacteria (3.05% vs. 1.36%) was higher in the CON group, whereas Firmicutes (15.83% vs. 26.09%) and Fusobacteria (11.11% vs. 34.99%) were higher in the PKE group. On day 28, the proportion of Bacteroidetes (44.37% vs. 36.07%) and Proteobacteria (23.19% vs. 0.47%) was higher in the CON group, whereas Firmicutes (9% vs. 14.16%), Synergistetes (3.15% vs. 15.53%), and Fusobacteria (16.67% vs. 29.22%) were higher in the PKE group. At the family level, on day 7, the proportion of *Bacteroidaceae* (65.56% vs. 30.32%) and *Peptostreptococcaceae* (8.61% vs. 4.22%) was higher in the CON group, whereas *Fusobacteriaceae* (11.11% vs. 34.99%) and *Lactobacillaceae* (0.83%vs. 14.78%) were higher in the PKE group (Fig. 4B). On day 28, the proportion of *Enterobacteriaceae* (22.75% vs. 0%) and *Enterococcaceae* (3.15% vs. 0%) was higher in the CON group, whereas *Synergistaceae* (3.15% vs. 15.53%), *Ruminococcaceae* (0% vs. 3.2%), *Prevotellaceae* (4.28% vs. 12.79%) and *Peptostreptococcaceae* (1.13% vs. 7.76%) were higher in the PKE group.

At the genus level, on day 7, the proportion of *Bacteroides* (65.56% vs. 30.37%) and *Clostridium*_XIX (5% vs. 0%) was higher in the CON group, whereas *Fusobacterium* (6.11% vs. 34.99%) and *Lactobacillus* (0.83% vs. 14.78%) were higher in the PKE group (Fig. 4C). On day 28, the proportion of *Bacteroides* (36.71% vs. 16.89%) and *Proteus* (22.75% vs. 0%) was higher in the CON group, whereas *Pyramidobacter* (2.93% vs. 14.16%), *Prevotellaceae*_unclassified (4.28% vs. 10.5%), *Prevotella* (0% vs. 2.28%), and *Clostridium*_XIX (9% vs. 18.26%) were higher in the PKE group. Overall, piglets showed a clear difference in taxa abundance between the two groups compared to sows, and the change in bacterial taxa was also significant at day 7 and day 28.

## Discussion

As interest in the gut microbiota increases, studies on the interaction between the host and microbes are being conducted in various fields. In the field of animal science, research into the relationship between livestock and intestinal microbes is ongoing, and studies aimed at improving performance and health through feed composition and additives are being reported continually [22, 23]. However, the effect of feeding sows with PKE, which is known to be high in fiber, on the gut microbiota of sows and their offspring remains elusive. Here, we show that PKE affects the gut microbiota of sows and, interestingly, confirmed that feeding of PKE to sows also affects the gut microbiota formation of their offspring.

In this study, we first checked whether there was a difference in the composition of the microbial community in sows and piglets through microbial diversity analysis. As a result, there was no difference in microbial diversity in sows, but we found that there was a significant difference between the PKE and CON groups in the beta-diversity analysis results in weaning-stage piglets. On day 7, there was no difference in diversity because the microbes were in the immature stage in the intestines of both groups, but in the weaning stage of piglets, intestinal microbes were colonized normally, and it seems that there was a significant difference in beta-diversity between the CON and PKE groups [24, 25]. This suggests that PKE feeding did not affect the microbial diversity of sows but did change the composition of microbial communities in piglets born to sows fed PKE. We confirmed that the type of diet can affect the formation of intestinal microbes in piglets by analyzing the diversity of microorganisms.

To further explore how the supplemental diet of PKE to lactating sow produced changes in the gut microbiota composition of sows and piglets, we sequenced the gut microbiota of sows and piglets and confirmed that dietary PKE changes the gut microbiota composition. It is also well known that the composition of maternal milk influences the formation of the gut microbiota of the offspring and therefore the maternal diet is important for both mother and offspring [26]. However, we discovered only subtle shifts in the gut microbiota composition in lactating sows between the CON and PKE groups, thereby implying a direct effect of the bacteria on sow metabolism rather than through the adjustment of the overall intestinal environment. Notably, the proportion of *Lactobacillus* in the gut microbiota of the group of sows fed PKE increased. *Lactobacillus* is known as one of the key genera inhabiting swine intestine [27, 28]. An increase in beneficial microorganisms such as *Lactobacillus* increases the production of mucin, which improves the intestinal barrier and is effective for feed efficiency [29]. Furthermore, from the overall study of the swine gut microbiota reported so far, it is clear that *Lactobacillus* in particular has a positive effect on the gut health of swine [30-32]. These findings suggest that dietary PKE for lactating sow may have a positive effect on the abundance of *Lactobacillus* present in the gut and may play a role in regulating gut conditions in which bacteria can indirectly produce beneficial metabolites.

Additionally, we identified changes in the gut microbiome composition of PKE-fed sows as well as their offspring. The composition of the gut microbiota on days 7 and 28 after birth showed that the abundance of *Fusobacterium* was higher in the PKE group compared to the CON group. *Fusobacterium* is common genus in the microbiome of piglets and an anaerobic, gram-negative bacterium whose main metabolite is butyric acid [33]. Although *Fusobacterium* has generally been reported to increase abundance in piglets with various intestinal disorders [34], the most recently published study showed an increased content of *Fusobacterium* members in the rectum of healthy piglets [33]. Therefore, the role of *Fusobacterium* in the gut microbiota of piglets cannot be interpreted in one direction with certainty. *Lactobacillus* showed higher abundance than the CON group at day 7. Since *Lactobacillus*, the most common genus in the piglet microbiome, is known to dominate in the healthy gut [35], this suggests that it may be beneficial for intestinal development and growth of piglets of the PKE group. The bacterium showing the biggest difference in gut microbiota composition at the weaning stage (day 28) was *Proteus*, which was found only in the CON group and is the most common opportunistic pathogen. This bacterium was significantly increased in the PECL (pectin+lipopolysaccharide-challenged piglets) group [36]. In the PKE group, *Prevotellaceae*_unclassified and *Prevotella* were relatively higher. *Prevotella* belonging to the *Prevotellaceae* family play an important role in decomposing polysaccharides and forming SCFAs, and several enzymes produced from *Prevotella* are known to be involved in polysaccharide (starch) degradation [37]. Also found was a higher relative abundance in the gut microbiota of *Prevotella* spp., which are reported to be essential for efficient digestion and absorption, and are positively correlated with average daily gain (ADG) by postweaning piglets [34, 38].

Our work reveals changes of the microbial composition in the gut microbiota during the lactation period in sows fed PKE and their piglets. Dietary PKE increased *Lactobacillus* abundance in sows. In piglets, *Lactobacillus* and *Prevotella*, which are known to have high abundance in the intestines of healthy piglets, were increased. These findings could be helpful in developing feeding strategies to promote the gut health of livestock. To fully understand the mechanisms by which the feeding of PKE causes changes in the gut microbiome for sows and their offspring, a more in-depth study is needed to investigate the interactions between the taxa identified here and PKE components, including fiber, and their overall impact on host metabolic health.
